# Novel *denovo TRPV4* mutation identified in a Chinese family with metatropic dysplasia inhibits chondrogenic differentiation

**DOI:** 10.1016/j.gendis.2023.05.008

**Published:** 2023-07-01

**Authors:** Ping Wei, Weizhe Shi, Tianying Nong, Caixia Xian, Xia Li, Zhaohui Li, Xin Li, Jianping Wu, Liyuan Shang, Fulong Xu, Yibo Xu, Hongwen Xu, Mingwei Zhu

**Affiliations:** a Guangzhou Institute of Pediatrics, Guangzhou Women and Children's Medical Center, Guangzhou Medical University, Guangdong Provincial Clinical Research Center for Child Health, Guangzhou, Guangdong 510623, China; b Department of Pediatric Orthopedics, Guangzhou Women and Children's Medical Center, Guangzhou Medical University, Guangdong Provincial Clinical Research Center for Child Health, Guangzhou, Guangdong 510623, China

Metatropic dysplasia (MD, MIM 156530) is a rare congenital bone dysplasia primarily characterized by severe platyspondyly with long and wide vertebral bodies and dumbbell deformity of the tubular bones. MD can be caused by heterozygous mutations in the gene encoding transient receptor potential vanilloid family member 4 (TRPV4). We identified a novel *de novo* mutation, c.2353 T  >  C, in the *TRPV4* gene, in a Chinese family with mild MD. Expression of mutant TRPV4 in HEK293 cells resulted in higher basal intracellular Ca^2+^ concentrations and endoplasmic reticulum redistribution. In addition, mutant TRPV4 inhibited the chondrogenic differentiation of ATDC5 cells. Overall, we concluded that the novel mutation c.2353 T  >  C in the *TRPV4* gene was the causative genetic lesion in this MD patient, whilst its pathogenicity might be partially attributed to the inhibition of chondrogenic differentiation.

Patients diagnosed with classic MD typically present with short postnatal stature, progressive kyphoscoliosis, joint movement restriction, and genu valgum. Additionally, radiographic phenotypes primarily include platyspondyly of the vertebral bodies, overfaced vertebral pedicles, widening and scalloping metaphyses of the tubular bones, shortness of the femoral neck, and delayed ossification of the carpal bones. In mild MD cases, kyphoscoliosis is not universal.^1^ *TRPV4* mutations were first found to cause MD in 2009.^2^ To date, over 50 mutations of *TRPV4* have been found to be associated with skeletal dysplasia, according to the Human Gene Mutation Database. TRPV4 can form a homo- or heterotetramer calcium-permeable nonselective cation channel that regulates intracellular Ca^2+^ concentrations.^3^ The absence or abnormal functioning of this channel can cause a variety of diseases, such as skeletal dysplasia and peripheral axonal neuropathy.^3^

The pedigree of a Chinese family with mild MD is shown in Figure 1A. The proband (II:2) was born naturally at full term and had no family history of skeletal dysplasia. After one year, the family found that she had a waddling gait due to a deformity of the lower limbs, which worsened over time. Comprehensive physical and radiographic examinations were performed at five years of age. Her weight was 13.6 kg (<–2 SD), whilst her height was 83 cm (<–6 SD). The patient walked in a typical waddling gait (Movie S1), with both knees presenting varus and flexion deformities (Fig. 1B). Meanwhile, the flexion, extension, and adduction of both hips were normal. However, the abduction of the left hip was normal, whilst that of the right hip was limited to 40°. No obvious abnormalities were observed in either upper limb. Although physiological curvature of the spine was observed, neither scoliosis nor kyphoscoliosis was observed (Fig. 1C). Muscle tone of the limbs was normal. Radiographic abnormalities included platyspondyly with irregular margins, a flattened acetabular roof, widening and scalloping metaphyses of the femur and tibia, and delayed ossification of the carpal bones (Fig. 1C–E). Therefore, she was diagnosed with mild MD. The patient's parents and family members exhibited no relevant clinical manifestations.

**Figure 1:**
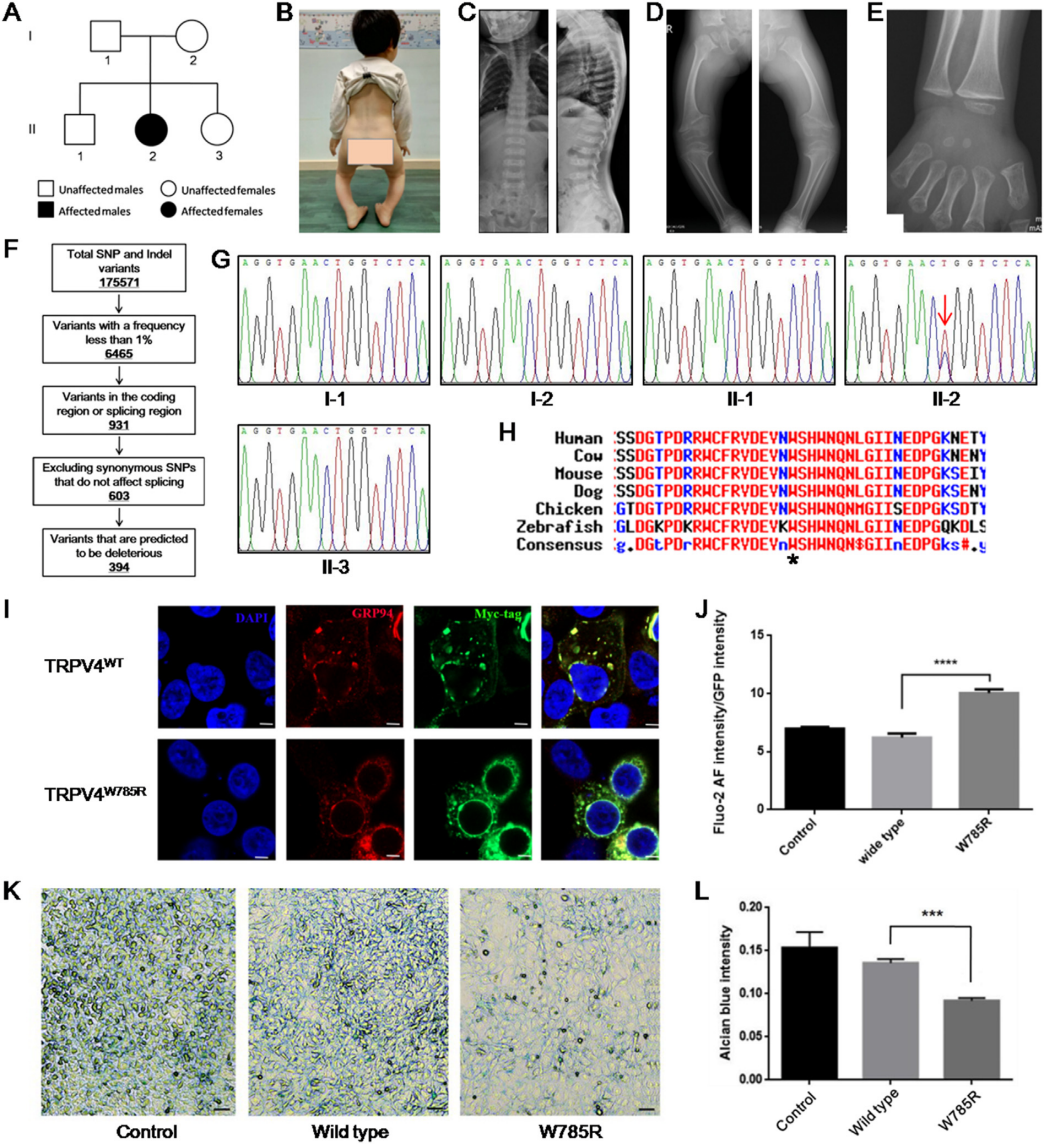
Novel *TRPV4* mutation identified in a Chinese family with mild MD inhibits chondrogenic differentiation. **(A)** The pedigree of the Chinese family with mild MD. II:2 is the patient. **(B)** Both knees of the patient present varus and flexion deformity. **(C)** X-radiograph of the patient's spine (left: front view; right: side view), showing platyspondyly with irregular margins. However, no scoliosis or kyphoscoliosis was observed. **(D)** X-radiograph of the patient's right and left lower limbs, showing flattened acetabular roof and widening and scalloping metaphyses of femur and tibia. **(E)** X-radiograph of the patient's right hand, indicating a delay in the ossification of carpal bones. **(F)** Schematic representation of the filtering process of WES data. **(G)** Sanger sequencing results from the blood gDNA of family members. The patient (II:2) carried the heterozygous mutation, c.2353 T  >  C in *TRPV4*. The red arrow points to the mutation site. **(H)** Alignment indicates that the amino acid affected by the mutation is evolutionarily conserved across species. **(I)** Subcellular localization of myc-hTRPV4^WT^ and myc-hTRPV4^W785R^ (red). Endoplasmic reticulum was indicated by GRP94 (green). The nucleus was indicated by DAPI (blue). Scale bar = 5 μm. **(J)** Quantitative analysis of basal intracellular Ca^2+^ concentration indicated by the fluorescence intensity of fluo-2 AM that was normalized by GFP. *n* = 4; ^∗∗∗∗^*P* < 0.0001. **(K)** ATDC5 cells were stained with Alcian blue to evaluate chondrocyte differentiation. **(L)** Quantitative analysis of the optical density of extracted Alcian blue from stained ATDC5 cells. *n* = 5; ^∗∗∗^*P* < 0.001.

The following is/are the supplementary data related to this article.

Supplementary video related to this article can be found at https://doi:10.1016/j.gendis.2023.05.008.

To identify the causative genetic lesion in the patient, we performed whole-exome sequencing of genomic DNA. As a result, a total of 175,571 genetic variants, including 155,060 SNPs (single nucleotide polymorphisms) and 20,511 indels (small insertion or deletion, <50 bp), were initially detected. After variant filtering, 325 SNPs and 69 indels remained (Fig. 1F). Among them, a heterozygous missense mutation in *TRPV4*, c.2353 T  >  C (NM_021625), was predicted to be deleterious with high scores (Table S1), which was not previously reported and was not included in reference gene databases such as the 1000 Genomes Project, Genome Aggregation Database, and NHLBI-ESP project. These findings were further validated by Sanger sequencing of the genomic DNA from family members (Fig. 1G). However, this mutation was only detected in the patient and not in her parents or other family members, indicating that this was a *de novo* mutation co-segregated with the skeletal dysplasia phenotypes. Overall, we reasoned that a missense mutation in *TRPV4* (c.2353 T  >  C) was the genetic lesion causing MD in the patient. However, according to the criteria of the American College of Medical Genetics and Genomics (ACMG), this mutation is classified as being of “uncertain significance” with evidence including PM2 (moderate pathogenic evidence) and PP3 (supporting pathogenic evidence).

Further experiments were conducted to confirm the pathogenicity of this novel mutation. The *TRPV4* (c.2353 T  >  C) mutation was predicted to result in the substitution of an evolutionarily conserved amino acid residue 785 tryptophan by arginine (p.W785R) (Fig. 1H). We constructed two vectors, N-myc-hTRPV4^WT^ and N-myc-hTRPV4^W785R^, to express wild-type and mutant human TRPV4 in HEK293 cells or mouse bone marrow mesenchymal stem cells, respectively. Although the transfection efficiency of N-myc-hTRPV4^W785R^ was lower than that of N-myc-hTRPV4^WT^, the molecular weight and expression level of hTRPV4^W785R^ were indistinguishable from those of hTRPV4^WT^ (Fig. S1). We also investigated the subcellular localization of mutant TRPV4 using immunofluorescence staining, with the results showing that hTRPV4^W785R^ was located in the endoplasmic reticulum, which was marked by GRP94 (glucose-regulated protein 94), similarly to hTRPV4^WT^. However, the overexpression of hTRPV4^W785R^ resulted in the redistribution of most of the endoplasmic reticulum around the nucleus (Fig. 1I; Fig. S2A).

We further investigated whether this mutation had resulted in an altered intracellular Ca^2+^ concentration. For this, the calcium fluorescent probe fluo-2 AM was used to detect basal intracellular Ca^2+^ concentrations. The results subsequently showed that overexpression of hTRPV4^W785R^ resulted in a higher basal intracellular Ca^2+^ concentration, thus indicating the enhanced activity of the mutant TRPV4 channel (Fig. 1J; Fig. S2B). Additionally, the overexpression of hTRPV4^W785R^ caused rounded cell morphology alongside low cell viability, which may have reflected the altered osmotic pressure of the cells due to excessive intracellular Ca^2+^.

Abnormal chondrogenesis is among the primary causes of skeletal dysplasia in MD. TRPV4 has previously been identified as a regulator of chondrogenic differentiation.^4^ We also investigated whether hTRPV4^W785R^ affected chondrocyte differentiation *in vitro*. ATDC5 cells infected with lentiviral vectors expressing hTRPV4^WT^ or hTRPV4^W785R^ were induced to differentiate into chondrocytes. Subsequently, Alcian blue staining highlighted that ATDC5 cells infected with Lenti-hTRPV4^W785R^ formed fewer cartilage nodules than cells infected with the control lentivirus or Lenti-hTRPV4^WT^ (Fig. 1K), which was further confirmed by quantification of Alcian blue staining intensity (Fig. 1L). hTRPV4^W785R^ had a similar inhibitory effect on the chondrogenic differentiation of mouse bone marrow mesenchymal stem cells (Fig. S2C). These results ultimately suggested that the mutant TRPV4 inhibited chondrogenic differentiation. However, the precise pathogenetic mechanism underlying *TRPV4* mutations that cause chondrodysplasia is not well understood. Intracellular Ca^2+^ signaling and extracellular Ca^2+^ balance both play critical roles in the normal development and maintenance of bone and cartilage. In 2008, Rock et al proposed a gain-of-function mechanism, since the mutations that they identified to cause skeletal dysplasia simultaneously resulted in an increased Ca^2+^ influx.^5^ However, many different mutations associated with skeletal dysplasia, including in-frame deletions and frameshift mutations, have since been identified, which makes it unlikely that all of them result in a gain-of-function. In addition, several *TRPV4* mutations that cause skeletal dysplasia do not elicit higher intracellular Ca^2+^ concentrations.^2^ Thus, whether elevated intracellular Ca^2+^ concentrations account for chondrodysplasia remains to be clarified.

In conclusion, we identified a novel mutation in *TRPV4*, c.2353 T  >  C (p.W785R), which caused MD. This mutation could elicit an increase in the basal intracellular Ca^2+^ concentration whilst inhibiting chondrogenic differentiation. Our findings expand the genetic etiology spectrum of MD and may be helpful for clinical genetic counseling and prenatal diagnosis of MD.

## Ethics declaration

The study was approved by the Human Ethics Committee of the Guangzhou Women and Children's Medical Center. Written informed consent was obtained from each participant or legal custodian.

## Author contributions

M.Z. and H.X. conceived and designed the experiments. P.W., T.N., C.X., X.L., Z.L., X.L., L.S., and Y.X. performed the experiments and analyzed the data. W.S., J.W., and F.X. collected the clinical data. All authors interpreted and discussed the results. M.Z. and P.W. wrote the manuscript. All authors read and approved the final manuscript.

## Conflict of interests

The authors declare no conflict of interests.

## Funding

This work was supported by the 10.13039/501100001809National Natural Science Foundation of China (No. 81972038 to M.Z.) and the 10.13039/501100003453Natural Science Foundation of Guangdong Province, China (No. 2023A1515010281 to M.Z.).
