# Supplementary material for: Novel denovo TRPV4 mutation identified in a Chinese family with metatropic dysplasia inhibits chondrogenic differentiation
Source: Genes Dis. 2023 Jul 1;11(3):101006. doi: 10.1016/j.gendis.2023.05.008 (PMC10825422; doi:10.1016/j.gendis.2023.05.008)
Supplement: Multimedia component 1 [file mmc1.docx]

**Materials and Methods**

**Patients and ethics**

The patient, a 5-year-old Chinese girl, was referred to Guangzhou Women and Children’s Medical Center for abnormal gait and malformation of both lower limbs. Physical and radiographic examinations were performed. The rest of her family is normal. The study protocols were approved by the Human Ethics Committee of the Guangzhou Women and Children’s Medical Center. Written informed consent was obtained from each participant or legal custodian.

**Genomic DNA preparation and whole-exome sequencing**

Genomic DNA (gDNA) was extracted from the peripheral blood of the patient with a Blood DNA Kit (Omega, USA) according to the manufacturer’s instructions. Whole-exome sequencing (WES) was performed at Novogene (Beijing, China) as previously described^1^. Simply, gDNA samples were randomly fragmented to an average size of 150~300 bp and subjected to DNA library construction. Then, the qualified libraries were sequenced with the Illumina HiSeq Xten System. The raw data were collected using Illumina Base Calling software (bcl2fastq). The Genome Analysis Toolkit (GATK v3.3.0) was employed to detect single nucleotide variants and indels (SNV/INDEL). ANNOVAR software was used to annotate these variants. Finally, all variants were further filtered following a pipeline: 1) exclusion of variants with a frequency greater than 1% in any of the four databases, including 1000g_all, esp6500siv2_all, gnomAd_ALL and gnomAD_EAS; 2) exclusion of variants that were not in the coding (exonic) region or splicing region (splicing site±10 bp); 3) exclusion of synonymous SNPs that were not predicted by dbscSNV to affect splicing; and 4) retention of variants that were predicted by at least two of four prediction tools (SIFT, Polyphen, MutationTaster, and CADD) to be deleterious and variants that were predicted to affect splicing.

**Variant verification**

The mutation in *TRPV4* was further confirmed by Sanger sequencing. The gDNA sequence covering the mutation site was amplified with the following primers: 5’-TGCTGAACCTGAGCAATGGC-3’ (forward) and 5’-CGATGGTCAGCTTAAA CAGGTCC-3’ (reverse). The qualified products were sequenced by Shanghai Sangon Biotech (Shanghai, China).

**Construction of expression plasmids and transfection of HEK293 or mouse BMSC cells**

The cDNA corresponding to the full-length wild type *TRPV4* (NM_021625) open reading frame was amplified from a commercial human *TRPV4* ORF clone (F122362, Youbao Biological, China). The cDNA was inserted between the SalI and BamHI restriction sites on the pBudCE4.1-EGFP-N-myc vector, and then sequenced^2^. The generated construct, hereafter N-myc-hTRPV4^WT^, expressed an N-terminal-myc tagged wild type human TRPV4 under CMV promoter and expressed EGFP under EF-1α promoter. EGFP was used as a reference to eliminate the impact of the differential transfection efficiency of vectors. To generate the N-myc-hTRPV4^W785R^ construct, N-myc-hTRPV4^WT^ was mutated with an M5 Site-Directed Mutagenesis Kit (Mei5bio, China). The following primers were used: forward 5’-GGTGAACCGGTCTCACTGGAACCAGAACTTGGGC-3’ and reverse 5’-TGAGACCGGTTCACCTCATCCACCCTGAAGCAC-3’.

Then, the empty vector, the N-myc-hTRPV4^WT^ construct and the N-myc-hTRPV4^W785R^ construct were transfected into HEK293 or mouse BMSC cells with Lipofectamine TM 3000 (Invitrogen, USA). Under 5% CO_2_ at 37°C, HEK293 cells were cultured in Dulbecco's modified Eagle's medium (DMEM) supplemented with 10% (v/v) fetal bovine serum (Gibco), 100 U/ml penicillin G, and 100 μg/ml streptomycin. C57BL/6 mouse BMSC cells were cultured in Mouse Mesenchymal Stem Cell Growth Medium from Cyagen Bioscience (MUXMX-90011, China).

**Western blot analysis**

After 48 h of culture after transfection, HEK293 cells were harvested and lysed on ice in RIPA buffer containing a protease inhibitor cocktail. The protein concentration was determined using a Pierce BCA Protein Assay kit (Thermo Fisher Scientific, USA). Then, Western blotting was performed according to standard procedures as previously described.^2^ The following primary antibodies were used: mouse anti-myc (1:1000, Cell Signaling Technology), rabbit anti-GFP (1:1000, Proteintech), and mouse anti-β-actin (1:2000, Cell Signaling Technology). HRP-conjugated anti-mouse antibody was used as the secondary antibody.

**Basal intracellular Ca^2+^ measurement**

Following transfection for 48 h, HEK293 or mouse BMSC cells were washed twice with Hank’s balanced salt solution (HBSS), and then loaded with the calcium fluorescent probe fluo-2 AM (AAT Bioquest, California) for 45 min at 37°C. Then, the cells were washed twice with HBSS to remove residual fluo-2 AM. The intensity of fluorescence was measured with a Varioskan LUX multimode microplate reader (Thermo Fisher Scientific, USA). The excitation wavelength for fluo-2AM is 340 nm, and the emission wavelength is 510 nm.

**Immunofluorescence and confocal imaging**

Transfected cells were fixed with 4% paraformaldehyde for 25 min and washed three times in PBS. Then, the cells were permeabilized in 0.5% Triton X-100/PBS for 10 min and subsequently blocked in 5% normal goat serum in PBS for 1 h at room temperature. The cells were then incubated with mouse anti-myc (1:1000, Cell Signaling Technology) and rat anti-GRP94 antibodies (1:4000, Abcam) at 4°C overnight. The secondary antibodies used were Alexa Fluor 549 goat anti-mouse IgG (1:1000) and Alexa Fluor 647 goat anti-rat IgG (1:1000). Finally, the cells were counterstained with DAPI (Vector Lab). Confocal images were captured on a Leica TCS SP8 laser scanning confocal microscope with a 60x oil objective.

**ATDC5 or mouse BMSC cells culture and chondrogenic differentiation**

ATDC5 cells, a murine chondrogenic cell line, were cultured in DMEM/Ham’s F-12 mixture medium (Gibco, USA) containing 10% fetal bovine serum. To induce chondrogenic differentiation, the medium was supplemented with 1% insulin-transferrin-sodium selenite (ITS) (Sigma, USA) and 50 μg/ml vitamin C (Sigma, USA) when the cell confluence reached approximately 90%. The medium was replaced once every 2 days. Mouse BMSC cells were cultured in Mouse Mesenchymal Stem Cell Growth Medium from Cyagen Bioscience (MUXMX-90011, China). To induce chondrogenic differentiation, the medium was supplemented with 1% insulin-transferrin-sodium selenite (ITS) (Sigma, USA) and 50 μg/ml vitamin C (Sigma, USA).

**Lentiviral vector and infection of ATDC5 or mouse BMSC cells**

Lentiviral vectors with an ubiquitin promoter driving either the wild type (Lenti-hTRPV4^WT^) or mutant TRPV4 (Lenti-hTRPV4^W785R^) were generated by GeneChem (Shanghai, China). For the overexpression experiments, ATDC5 or mouse BMSC cells were seeded at a density of 3×10^4^ cells/well in a 12-well plate. When confluence reached approximately 20%, the cells were infected with the lentivirus construct at a multiplicity of infection (MOI) of 100.

**Alcian blue staining**

The cells were washed twice with PBS, fixed with 4% paraformaldehyde for 20 min, and then stained with Alcian blue (Sigma, USA) solution for 15 min at room temperature. Images were captured with a Leica DM4 microscope. To quantify Alcian blue staining, the cells were scraped off and then incubated with 6 M guanidine-HCl for 8 h at 25 °C to extract Alcian blue. The absorbance of the supernatant was measured at 620 nm with a Multiskan GO spectrophotometer (Thermo Fisher Scientific, USA).

**Statistical analysis**

An unpaired Student’s *t*-test was used to evaluate the significance of the experiments. *P* values<0.05 were considered to be significant.

**Supplemental References**

1. Li X, Shi W, Ding X, et al. Identification of a novel TBX5 mutation in a Chinese family with rare symptoms of Holt-Oram syndrome. *Heliyon*. 2022; 8(11):e11774.

2. Xian C, Zhu M, Nong T, et al. A novel mutation in ext2 caused hereditary multiple exostoses through reducing the synthesis of heparan sulfate. *Genet Mol Biol*. 2021; 44(2):e20200334.

**Supplemental Figures**


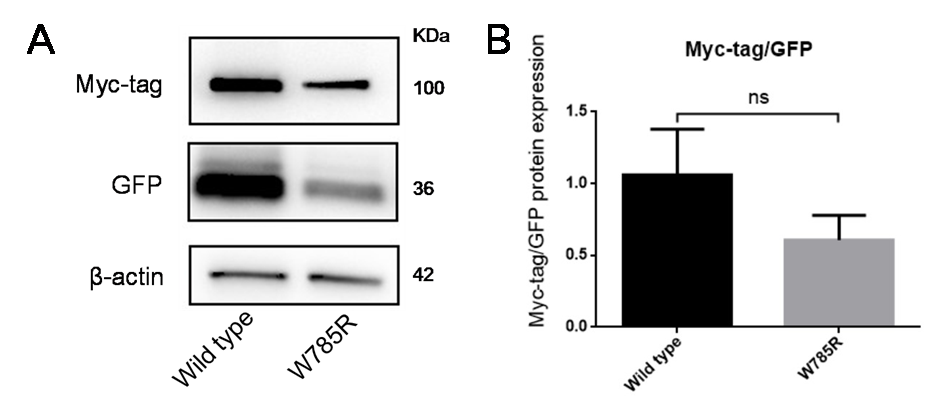


**Figure S1. The expression of hTRPV4^WT^ and hTRPV4^W785R^ in HEK293 cells.** The molecular weight (A) and expression level (B) of hTRPV4^W785R^ were indistinguishable from that of hTRPV4^WT^ in HEK293 cells. Note the transfection efficiency of N-myc-hTRPV4^W785R^ was lower as indicated by the lower expression of GFP. β-actin was used as the control. n.s, no significance.


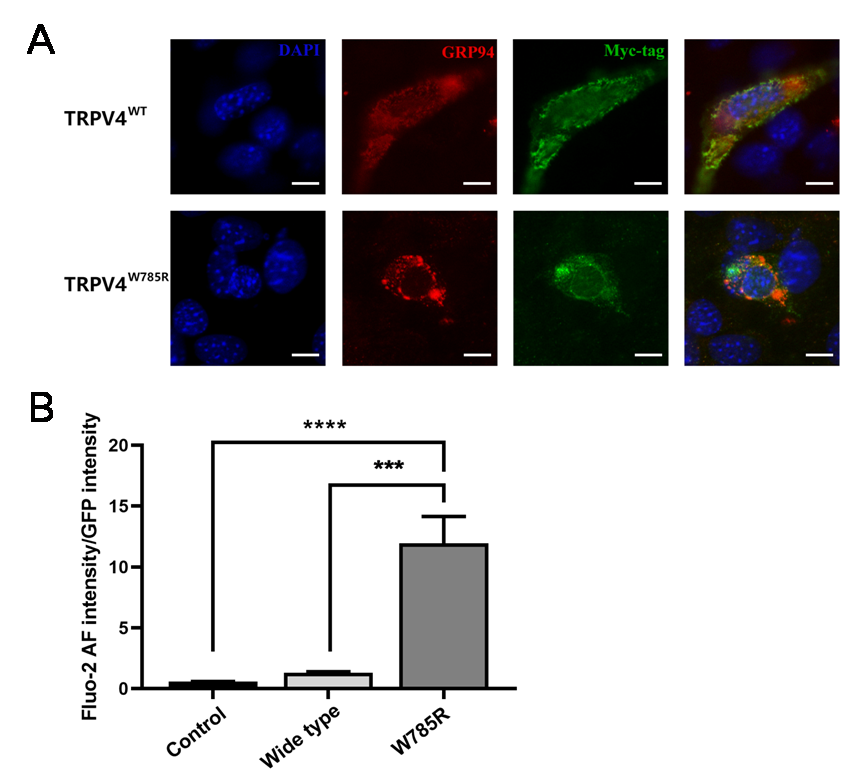


**Figure S2. The expression of hTRPV4^WT^ and hTRPV4^W785R^ in mouse BMSCs.** (A) Subcellular localization of myc-hTRPV4^WT^ and myc-hTRPV4^W785R^ (red). Endoplasmic reticulum was indicated by GRP94 (green). Nucleus was indicated by DAPI (blue). Scale bar = 10 μm. (B) Quantitative analysis of basal intracellular Ca^2+^ concentration indicated by the fluorescence intensity of fluo-2 AM that was normalized by GFP. n=3, *** *P*<0.001, **** *P*<0.0001.


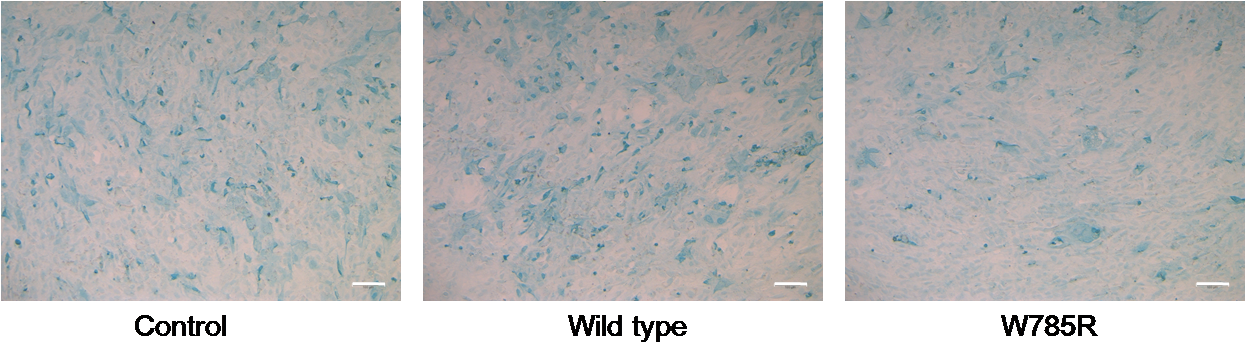


**Figure S3. hTRPV4^W785R^ inhibited chondrogenic differentiation of mouse BMSCs**. Alcian blue staining was used to evaluate chondrogenic differentiation. Scale bar = 100 μm.

**Supplemental Tables**

**Table S1.** Deleterious prediction of *TRPV4* c.2353T>C mutation.

| **Position** | **Gene** | **Transcript** | **Nucleotide change** | **Amino acid change** | **SIFT** | **Polyphen2** | **Mutation Taster** |
| --- | --- | --- | --- | --- | --- | --- | --- |
| Chr12: 110222226 | *TRPV4* | NM_021625 | c.T2353C | p.W785R | D | D | D |

Note: Chr, chromosome; SIFT, Sorting Intolerant From Tolerant; D, deleterious.
